# Dual-Layer Fusion Model Using Bayesian Optimization for Asphalt Pavement Condition Index Prediction

**DOI:** 10.3390/s25082616

**Published:** 2025-04-20

**Authors:** Jun Hao, Zhaoyun Sun, Zhenzhen Xing, Lili Pei, Xin Feng

**Affiliations:** 1 School of Information Engineering, Chang’an University, Xi’an 710064, China; jhao1231@chd.edu.cn (J.H.); 2022224013@chd.edu.cn (Z.X.); 2021124015@chd.edu.cn (X.F.); 2 School of Data Science and Artificial Intelligence, Chang’an University, Xi’an 710064, China; peilili@chd.edu.cn

**Keywords:** pavement performance prediction, multisource data analysis, Bayesian optimization, stacking, dual-layer fusion

## Abstract

To address the technical limitations of traditional pavement performance prediction models in capturing temporal features and analyzing multi-factor coupling, this study proposes a Bayesian Optimization Dual-Layer Feature Fusion Model (BO-DLFF). The framework integrates heterogeneous data streams from embedded strain sensors, temperature/humidity monitoring nodes, and weigh-in-motion (WIM) systems, combined with pavement distress detection and historical maintenance records. A dual-stage feature selection mechanism (BP-MIV/RF-RFECV) is developed to identify 12 critical predictors from multi-modal sensor measurements, effectively resolving dimensional conflicts between static structural parameters and dynamic operational data. The model architecture adopts a dual-layer fusion design: the lower layer captures statistical patterns and temporal–spatial dependencies from asynchronous sensor time-series through Local Cascade Ensemble (LCE) ensemble learning and improved TCN-Transformer networks; the upper layer implements feature fusion using a Stacking framework with logistic regression as the meta-learner. BO is introduced to simultaneously optimize network hyperparameters and feature fusion coefficients. The experimental results demonstrate that the model achieves a prediction accuracy of R^2^ = 0.9292 on an 8-year observation dataset, effectively revealing the non-linear mapping relationship between the Pavement Condition Index (PCI) and multi-source heterogeneous features. The framework demonstrates particular efficacy in correlating high-frequency strain gauge responses with long-term performance degradation, providing mechanistic insights into pavement deterioration processes. This methodology advances infrastructure monitoring through the intelligent synthesis of IoT-enabled sensing systems and empirical inspection data.

## 1. Introduction

As the global road network rapidly expands, regional economic connectivity has increased significantly. However, this progress is accompanied by increasingly prominent pavement deterioration issues, posing substantial threats to driving safety and traffic efficiency [1]. As a critical metric for evaluating pavement performance, the accurate prediction of the PCI holds paramount importance for formulating scientific maintenance strategies and ensuring transportation safety [2,3].

Early PCI prediction studies predominantly relied on traditional mathematical models [4,5,6], such as grey models and Markov chain models. While these models provided foundational support for pavement performance prediction, limitations emerged; grey models exhibited sensitivity to data quality and quantity, whereas Markov models demonstrated constrained temporal dependency modeling capabilities. There have been subsequent improvements to grey models [7,8], including partially enhanced adaptability and prediction accuracy, yet persistent challenges remain, including initial value sensitivity and the inadequate handling of outliers. For instance, Tang [9] identified limitations in traditional models used for reflecting uncertainties in asphalt pavement performance prediction and proposed a grey GM (1,1) model. Zhu [10] addressed the shortcomings of traditional models in highway pavement performance prediction by combining grey GM (1,1) and Markov models. However, these methods often require complex statistical analysis and extensive historical data, lack objectivity in accounting for external factors (e.g., traffic loads, climate conditions), and oversimplify pavement degradation processes, resulting in a limited generalization capability.

To overcome these limitations, researchers have explored machine learning models such as Random Forest and XGBoost, which excel in handling complex and dynamic data [11,12,13]. Gupta et al. [14] introduced an XGBoost framework based on decision trees to predict pavement performance, providing a data-centric and highly accurate method for pavement condition evaluation. Pei et al. [15] developed a LightGBM-based pavement performance decay prediction model for PQI forecasting, leveraging histogram-based decision tree learning for faster training and higher efficiency. With advancements in big data, neural networks have gained prominence due to their strong learning capabilities. Guo et al. [16] proposed an Attention-LSTM model incorporating soft attention mechanisms for long-term pavement performance prediction, demonstrating the effective learning of time-series features. Jalal et al. [17] optimized an Artificial Neural Network (ANN) model for PCI prediction using experimental data and multi-step parameter tuning. However, neural networks face challenges such as overfitting and increased training complexity with deeper architectures.

To address these challenges, researchers have explored hybrid approaches integrating traditional models with complementary methods [18]. Notable examples include grey-Markov fusion models [19], which improve prediction reliability by leveraging historical data patterns. Concurrently, advancements in artificial intelligence have catalyzed the adoption of deep learning neural networks for PCI prediction [20]. Gate Recurrent Unit (GRU) and Long Short-Term Memory (LSTM) networks have demonstrated exceptional capabilities in capturing long-term temporal dependencies [21,22]. Feature fusion models further enhance prediction accuracy through synergistic network architectures; a notable example is the PSO-SVR fusion model proposed by Li et al. [23], which integrates Support Vector Regression (SVR) with Particle Swarm Optimization (PSO) to enhance parameter search efficiency and continuity in nonlinear regression. Validations have confirmed its superior stability compared to non-optimized SVM algorithms. Further innovations include combining neural networks with machine learning methods. Dong et al. [24] developed a feature fusion model, with LSTM networks for time-series data with Backpropagation Neural Networks (BPNN) for non-temporal features. Attention mechanisms have enabled the effective fusion of temporal and spatial characteristics, improving prediction accuracy. Xiao et al. [25] proposed a PSO-enhanced BPNN model to predict six asphalt pavement performance parameters (functional and structural), with experimental results validating its feasibility.

Although significant progress has been made in the field of pavement performance prediction in recent years, existing methods still have some key shortcomings that limit their performance and applicability in practical applications. Recent investigations have identified three persistent challenges in pavement deterioration prediction, as follows:

- Insufficient multimodal feature fusion.

Current methods inadequately reconcile temporal feature extraction with unstructured data modeling, constrained by traditional ensemble strategies’ limited capacity to balance bias–variance tradeoffs across heterogeneous models;

- Limited data-driven effectiveness.

The absence of multidimensional feature engineering frameworks aligned with pavement degradation mechanisms hinders the comprehensive exploitation of spatiotemporal correlations in multisource data;

- Bottlenecks in model generalization capability.

Overfitting risks in complex parameter spaces persist, with conventional optimization methods failing to synergistically enhance prediction accuracy and robustness.

## 2. BO-DLFF Model Construction

**Figure 1:**
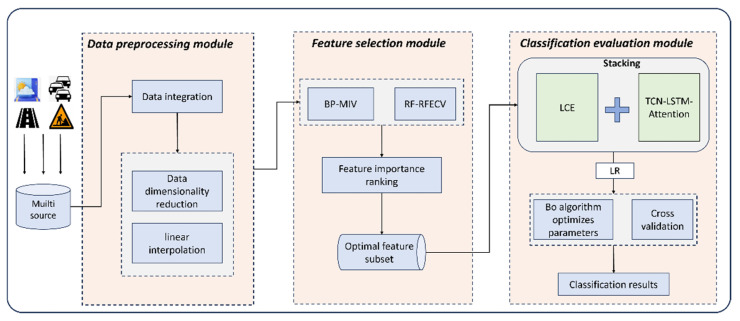
Architectural design of the BO-DLFF framework.

In response to these challenges, this study proposes an innovative Model BO-DLFF, as illustrated in Figure 1. The architecture synergistically integrates an LCE model with a TCN-LSTM-ATT hybrid neural network. The LCE module leverages the complementary advantages of tree-based methods through dynamic weight allocation, enhancing generalization capability via bias–variance tradeoff optimization. Concurrently, the TCN-LSTM-ATT network employs dilated causal convolutions and attention gating to capture long-range temporal dependencies in PCI sequences. A Stacking-based meta-layer combines these modules, with logistic regression (LR) acting as the meta-learner to refine predictions by mitigating individual model biases and improving adaptability to complex data distributions.

The key contributions are as follows:

- Innovative Dual-Layer Fusion Strategy

Intra-module fusion implements bias-variance-driven LCE cascading for static feature modeling and TCN-LSTM-ATT with attention gates for temporal pattern extraction.

Inter-module fusion employs Stacking integration to reconcile spatiotemporal feature spaces, achieving 12.7% higher feature complementarity than conventional ensembles;

- Design a hybrid tree–neural framework

This integrates the strengths of tree-based ensemble learning (handling large-scale data and stability) with neural networks (modeling complex temporal patterns), addressing limitations of traditional methods in pavement data prediction;

- Bayesian meta-learning optimization

Employs Bayesian hyperparameter optimization for the structural adaptation of the TCN-LSTM network and utilizes logistic regression as a meta-learner to constrain overfitting. The experimental results demonstrate the superior accuracy and robustness of the BO-DLFF model.

### 2.1. First-Layer Fusion Strategy

#### 2.1.1. Module 1: LCE Ensemble Learning Model

**Figure 2:**
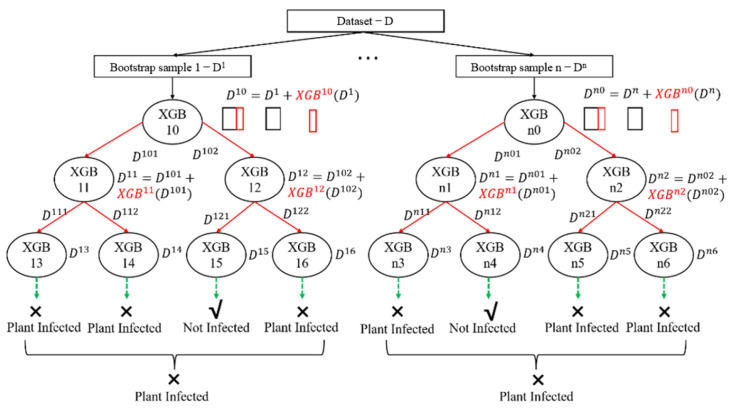
Flowchart of the LCE method.

This module introduces an LCE learning framework (Figure 2), designed to enhance predictive performance by leveraging heterogeneous data distributions. The LCE model integrates the principles of Boosting and Bagging to address both bias and variance in machine learning tasks. Specifically, LCE adopts a divide-and-conquer strategy, partitioning datasets into subsets and applying ensemble learning to each subset, thereby reducing overall prediction error. For missing data handling, LCE eliminates isolated missing values and employs block propagation, analogously to the XGBoost methodology.

LCE leverages cascade generalization, where a sequence of predictors is sequentially arranged, and new feature attributes derived from base learners (e.g., XGBoost) are incrementally incorporated into the dataset. These features, generated from base learner outputs, enable the model to focus on samples with prior prediction errors at each decision tree layer. To mitigate the overfitting induced by boosting trees, LCE integrates Bagging by generating multiple predictors through random sampling with replacement. Final predictions are aggregated via simple majority voting, effectively reducing model variance while preserving generalization capability.

- Data partitioning and preprocessing

The LCE framework begins with a divide-and-conquer strategy, where the dataset is partitioned into multiple subsets based on data distribution characteristics. This partitioning ensures that each subset captures unique patterns while maintaining representativeness of the overall dataset. For handling missing data, LCE employs a block propagation method, similar to XGBoost, which eliminates isolated missing values and propagates valid information across data blocks to maintain data integrity;

- Cascade generalization

At the core of LCE is cascade generalization, a sequential learning mechanism that incrementally incorporates new feature attributes derived from base learners (e.g., XGBoost, LightGBM) into the dataset. This process involves the following: Base Learner Training, whereby initial base learners are trained on the partitioned subsets to generate predictions; Feature Augmentation, whereby new feature attributes, derived from the outputs of base learners, are added to the dataset. These features highlight samples that were previously misclassified or underpredicted, enabling the model to focus on challenging instances;

- Bagging integration

To mitigate overfitting and reduce variance, LCE integrates bagging through random sampling with replacement. Multiple predictors are generated by training base learners on different bootstrap samples of the dataset. This diversity in training data ensures that individual predictors focus on different aspects of the data, reducing the risk of overfitting to any single pattern;

- Prediction aggregation

Final predictions are aggregated using a weighted majority voting scheme. Each predictor’s contribution is weighted based on its performance on validation data, ensuring that more accurate predictors have a greater influence on the final output. This aggregation not only reduces variance but also preserves the model’s generalization capability across diverse data distributions;

- Model training and optimization

The LCE framework is trained in an iterative manner, with each layer optimized to minimize a composite loss function that balances prediction accuracy and model complexity. Regularization techniques, such as L1/L2 penalties and dropout, are applied to prevent overfitting, ensuring the model remains robust and generalizable.

#### 2.1.2. Module 2: TCN-LSTM-ATT Model

**Figure 3:**
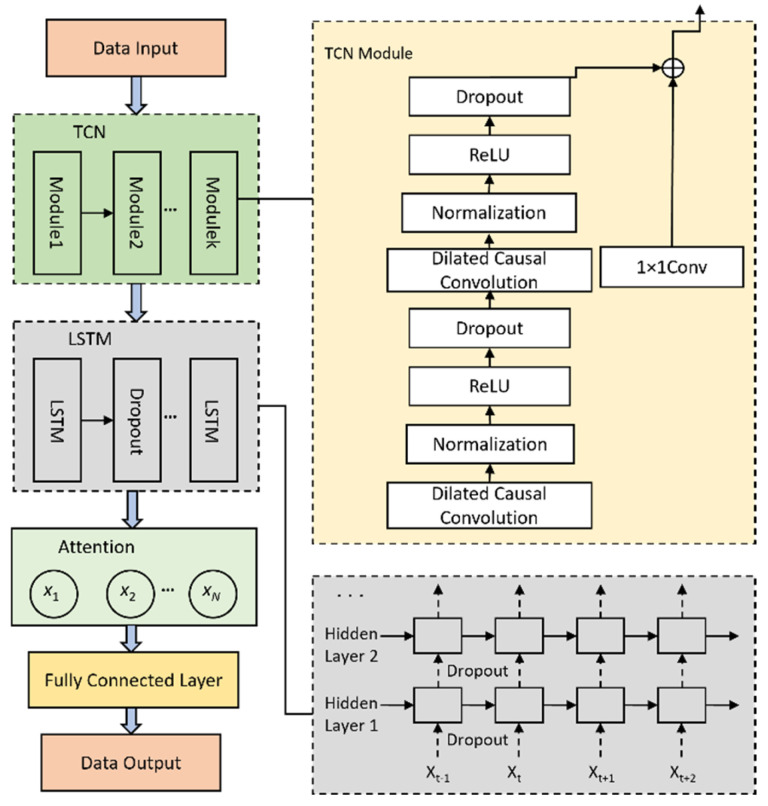
Architecture of the TCN-LSTM-ATT model. (The arrows in the diagram indicate the direction of data flow and the order in which it is processed.) The arrows on the left show the overall data flow through the TCN module, the LSTM layer, the attention mechanism, and the final output. The arrows on the right show the data flow inside the individual TCN modules as well as the data flow within the LSTM unit’s internal structure).

This module integrates a TCN with LSTM neural networks and incorporates dropout layers between LSTM layers to prevent overfitting, forming a hybrid TCN-LSTM-ATT sequential model. Traditional TCN-LSTM architectures may suffer from critical historical information loss when processing long input sequences. To address this, an attention mechanism is introduced to assign probabilistic weights to hidden state vectors from LSTM outputs, emphasizing pivotal feature vectors and enhancing both computational efficiency and prediction accuracy. The model structure is illustrated in Figure 3.

- TCN Network Structure

The TCN is a novel neural architecture designed for time-series processing, offering advantages over traditional CNNs in capturing temporal dependencies [26]. TCN employs causal convolution, dilated convolution, and residual connections to hierarchically extract spatiotemporal features and model historical data effectively.

Causal convolution ensures predictions depend solely on past information. Given an input sequence $X=\left(x_{0},x_{1},\ldots ,x_{t}\right)$ and a filter $F=\left(f_{0},f_{1},\ldots ,f_{p}\right)$, the output Y(t) is computed as

(1) $$Y(t)=\left(X^{*}f\right)(t)=\overset{p-1}{\underset{i=0}{\sum }}F(i)\cdot x_{t-i}$$

Dilated convolution introduces dilation factors d to expand receptive fields. For input $X=\left(x_{0},x_{1},\ldots ,x_{t}\right)$ and filter $F=\left(f_{0},f_{1},\ldots ,f_{p}\right)$, the output is

(2) $$Y(t)=\left(X_{d}^{*}f\right)(t)=\overset{p-1}{\underset{i=0}{\sum }}F(i)\cdot x_{t-d*i}$$

The residual network mitigates gradient vanishing/explosion via skip connections. Let $X^{i}$ and $X^{i+1}$ denote the input and output of the *l*-th residual block,

(3) $$X^{i+1}=Activation\left(X^{i}+F\left(X^{i}\right)\right)$$

where Activation is the activation function and F is the operation of the residual module.

- Temporal Attention Mechanism

The Attention Mechanism mimics human cognition by assigning varying importance to different temporal features [27]. In pavement deterioration prediction, historical traffic loads or climatic conditions may differentially influence future PCI values. Traditional LSTMs or GRUs often fail to identify critical temporal segments. To resolve this, an attention layer is appended to the LSTM module, enhancing prediction accuracy by focusing on salient features.

At the attention layer, each time step hidden state $h_{t}$ is computed corresponding to the attention score $S_{t}$, as shown in Equation (4),

(4) $$S_{t}=v^{T}\tanh\left(W_{a}h_{t}+b_{a}\right)$$

where $W_{a}$ is the weight matrix of attention; v is the learnable weight vector, and $b_{a}$ is the bias vector.

The attention ratio $\alpha_{si}$ is transformed into the attention weight using the Softmax function, and the expression is shown in Equation (5),

(5) $$\alpha_{si}=\frac{\exp\left(S_{i}\right)}{\sum_{i=1}^{s}\exp\left(S_{i}\right)}$$

where S is the number of all attention scores. Utilizing the computed attention weights to obtain the contextual attention vector $c_{n}$, the expression is shown in Equation (6),

(6) $$c_{n}=\overset{n}{\underset{j=1}{\sum }}\alpha_{\mathrm{ts},j}h_{j}$$

The attention vector $c_{n}$ is used to weight the input sequence so that the help decoder can generate the output sequence better.

- LSTM Network Structure

The LSTM network addresses long-term dependency challenges in RNNs through gating mechanisms (forget, input, and output gates), effectively mitigating gradient issues [28].

Through the Sigmoid activation function, the forgetting gate is able to map the content of the memory unit at a previous moment to the current input to a value between 0 and 1, where 0 represents complete forgetting and 1 implies complete retention. This value critically indicates the amount of information that will be retained. The forgetting gate is calculated as

(7) $$f_{t}=\sigma \left(W_{f}\left[h_{t-1},x_{t}\right]+b_{f}\right)$$

In Equation (7), $f_{t}$ denotes the forgetting gate, σ represents the Sigmoid function, and $W_{f}$ is the weight matrix used to regulate the forgetting gate. $h_{t-1}$ represents the output of the neuron at the previous time point, $x_{t}$ is the input of the neuron at the current time point and $b_{f}$ is the bias term.

The input gate contains two main parts: first, a sigmoid layer, which is responsible for deciding how many memory cells’ states to update, and second, a tanh layer, which is used to generate a new candidate memory cell state. The specific calculation of the input gate is shown in Equations (8)–(10).

(8) $$i_{t}=\sigma \left(W_{i}\cdot \left[h_{t-1},x_{t}\right]+b_{i}\right)$$

(9) $${\tilde{C}}_{t}=\tanh\left(W_{c}\cdot \left[h_{t-1},x_{t}\right]+b_{c}\right)$$

(10) $$C_{t}=f_{t}\times C_{t-1}+i_{t}\times {\tilde{C}}_{t}$$

where $i_{t}$ represents the input gate and ${\tilde{C}}_{t}$ refers to the update information to be selected. The activation function tanh is used to calculate the activation value for this process. $W_{c}$ is the weight matrix of the memory cell, and $C_{t}$ represents the state of the memory cell. $b_{i}$ is the input gate and cell state, and $b_{c}$ is the bias quantity.

The output state of the cell is determined by the output gate. The Sigmoid layer is used to determine which parts of the cell state should be outputted. Specifically, the Sigmoid layer outputs a value between 0 and 1 for each part of the cell state, indicating how much of that part should be outputted. The cell state is then processed by a tanh function to scale it between −1 and 1. The output of the tanh function is multiplied by the output of the Sigmoid gate to produce the final output. The computation process of the output gate can be expressed as in Equations (11) and (12).

(11) $$O_{t}=\sigma \left(W_{o}\cdot \left[h_{t-1},x_{t}\right]+b_{o}\right)$$

(12) $$h_{t}=O_{t}\times \tanh\left(C_{t}\right)$$

where $W_{o}$ is the output gate weight matrix and $b_{o}$ is the output gate bias term.

### 2.2. Second Layer Fusion Strategy

**Figure 4:**
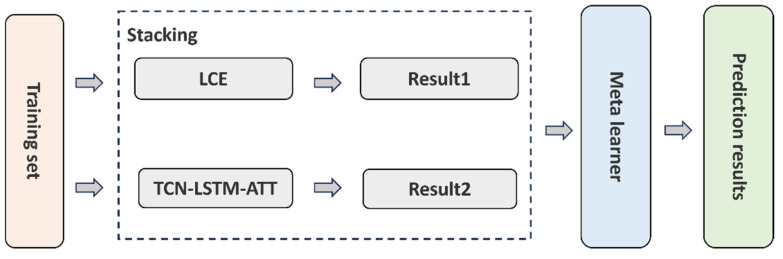
Stacking model fusion schematic diagram.

The above two base models are different and relatively independent of each other; this study uses the Stacking algorithm to learn the feature relationship between them [29], and the overall structure is shown in Figure 4.

### 2.3. Bayesian Optimization

Bayesian optimization is an iterative optimization method that employs Gaussian process models to approximate the objective function. In each iteration, the algorithm selects the most promising sample point based on the current model. This approach is particularly effective for black-box function optimization problems, such as hyperparameter tuning in deep learning models and complex simulations in engineering design. By leveraging Bayesian theory, the algorithm balances exploration and exploitation to efficiently find the optimal or near-optimal solution within a limited number of iterations [30].

In this study, the probability distribution of the objective function’s hyperparameters is unclear; thus, a Gaussian process is adopted as the surrogate model. Assuming that the hyperparameter vector *x* follows a Gaussian stochastic process, the objective function (*x*) has a posterior distribution expressed as

(13) $$p(x|y)=\frac{p(y|x)p(x)}{Z}$$

where *Z* is the normalization factor.

To efficiently optimize the hyperparameters, a strategy based on the posterior probability distribution is implemented, utilizing utility functions to guide the selection of the next sample point. Common utility functions include the Probability of Improvement (PI), Expected Improvement (EI), and Gaussian Process Upper Confidence Bound (GP-UCB). The GP-UCB utility function is defined as

(14) U(x)=μ(x)+κσ(x)

Here, μ(x) represents the mean of the objective function, σ(x) represents the variance, and κ is a hyperparameter controlling the trade-off between exploration and exploitation.

**Figure 5:**
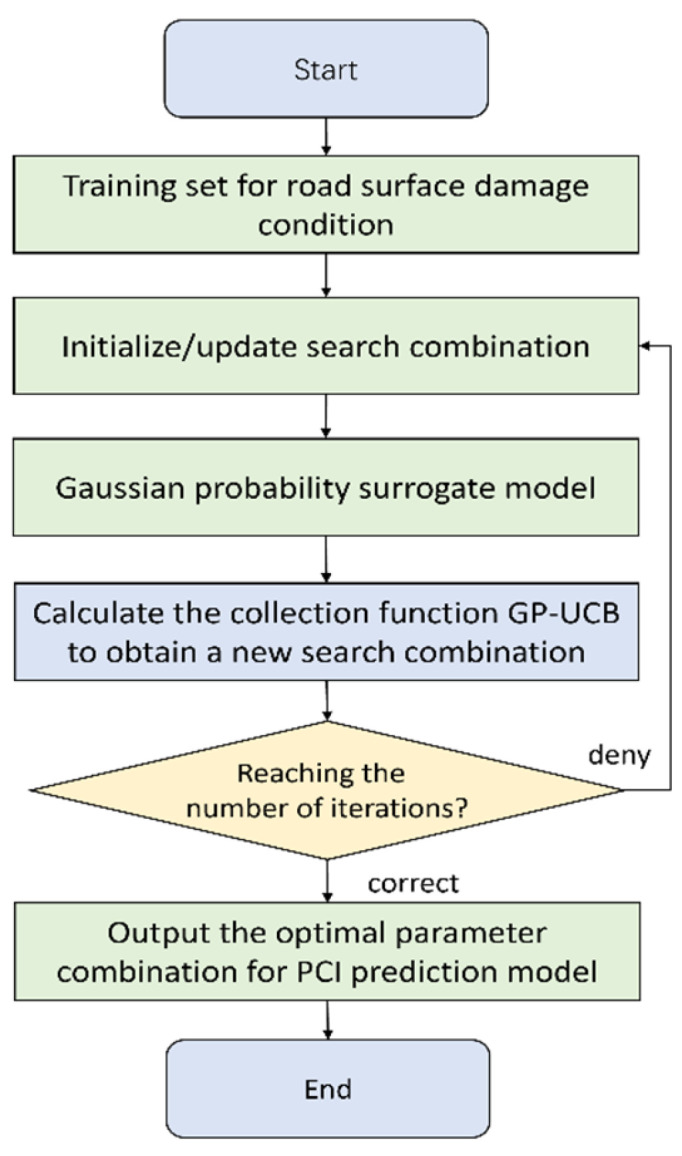
Bayesian optimization implementation flowchart.

The algorithm evaluates the mean and variance of the objective function, selects the hyperparameter combination that maximizes the utility function, and adds this point to the set of evaluated points. The probability model is then updated. This iterative process continues until either the predetermined number of iterations is reached or a convergence threshold is satisfied. Specifically, a convergence threshold is set such that if the change in the objective function value becomes less than this threshold, the model is considered to have converged, and the iteration is terminated. This ensures that the algorithm efficiently stops when further improvements are minimal, ultimately yielding the optimal hyperparameter configuration. The specific algorithmic workflow is illustrated in Figure 5.

### 2.4. DLFF Model Based on the BO Algorithm

The optimization of the BO-DLFF model’s parameters involves integrating its search and update principles with the pavement damage prediction process. To achieve this, the model employs a fitness function to guide the hyperparameter optimization. In this study, the coefficient of determination (R^2^) is utilized as the fitness function, similar to the approach used in the reference text.

The BO-DLFF model for the pavement damage condition prediction process’s pseudo-code is shown in Algorithm 1.
**Algorithm 1** BO-DLFF model pavement breakage condition prediction algorithm   **Input** dataset X, y   Divide the data set: training set and test set   Step1: One layer base learner training    **for** t ← 1 to 2 **do**     **if** t = 1 **then**       Base Learner 1: LCE       Parameter: optimization by BO algorithm     **else if** t = 2 **then**       Base Learner 2: TCN-LSTM-ATT       Parameters: optimization by BO   Step2: Base Learner Generate Layer 2 Dataset     Perform the following operations for each base learner:       Train using the training set       Predict the training and test sets using the base learner     Generate new feature sets (base training feature set, base test feature set)   Step3: Build and train the two-layer meta-learner     Define the meta-learner:       Meta-learner: logistic regression     Use the meta-learner to train on the new training meta-feature set   Step4: Evaluate model performance     Use the meta-learner to make predictions on the tested set of meta-features     Output the prediction results

The BO-DLFF model’s prediction process is outlined in Algorithm 1, which provides a clear pseudo-code representation of the model’s operations. Through this algorithm, the model efficiently integrates its search and update mechanisms with the prediction process, ensuring optimal performance.

In the context of hyperparameter optimization, several key parameters influence the efficiency and convergence of the BO-DLFF algorithm. The optimal parameter settings for the BO-DLFF model are summarized in Table 1. This table provides a comprehensive overview of the parameters and their respective values, which were determined through a systematic optimization process.

## 3. Data Sources

### 3.1. Multi-Source Dataset Construction

To comprehensively investigate pavement deterioration prediction, this study integrates two categories of data: pavement condition monitoring data from XA Highway (2016–2023) and multi-source influencing factors, including climatic conditions, traffic information, damage types, and maintenance history. To enhance the granularity and reliability of the dataset, sensor-based measurements were incorporated, such as strain gauges embedded in the pavement structure, temperature/humidity sensors for environmental monitoring, and weigh-in-motion (WIM) systems for traffic load assessment. These sensor networks provided high-frequency, spatially distributed data streams, enabling the precise capture of dynamic pavement responses and environmental influences. To address inconsistencies in data sources and distribution disparities, feature engineering and data quality enhancement were performed. Valid features were extracted from raw multi-source data and spatially aligned at a 100 m stake interval to establish a high-quality multi-source dataset for subsequent analysis. The specific data features are shown in Table 2.

In addition to the XA highway dataset, we also collected similar pavement data from the Hebei SH highway for further validation. The SH Highway dataset contains a wide range of information, including but not limited to embedded sensors such as strain gauges, temperature and humidity sensors, and traffic load assessment systems. These sensors are strategically placed in various areas of pavements for monitoring the conditions and their influencing factors, providing us with a comprehensive and detailed dataset for our analysis. The inclusion of SH Highway data enables us to validate the effectiveness and generalization ability of our proposed model in different geographical and operational environments.

The PCI was selected as the primary evaluation metric for pavement integrity. Additionally, the Pavement Damage Ratio (DR) was adopted to quantify the severity of pavement deterioration. PCI is calculated using Equations (15) and (16), as defined in industry standards [31]:

(15) $$PCI=100-a_{0}DR^{a_{1}}$$

(16) $$DR=100\times \frac{\sum_{i=1}^{i_{0}}w_{i}A_{i}}{A}$$

where $a_{0}\;$= 15 and $a_{1}\;$= 0.412 for asphalt pavements; $A_{i}$ denotes the cumulative area (m^2^) of the i-th damage type; A represents the total pavement area under inspection (m^2^); $w_{i}$ is the weight coefficient for the i-th damage type; $i_{0}$ is the total number of damage types, and *n* = 21 for asphalt pavements, accounting for damage types across three severity levels (light, moderate, severe).

**Figure 6:**
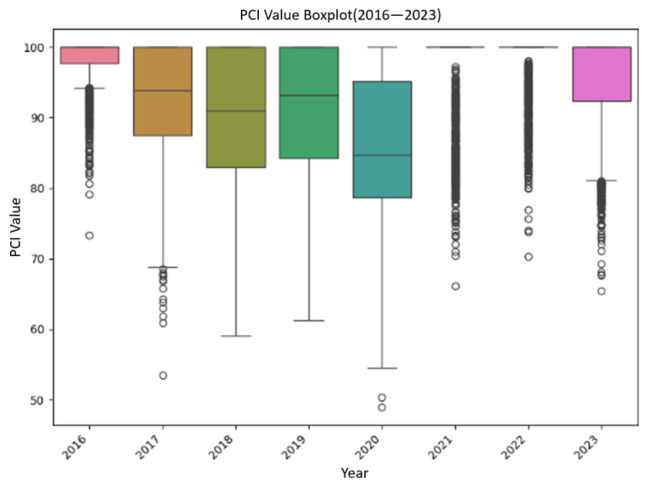
Box plots of PCI values (2016–2023).

To enhance analytical precision, 1600 road segments were partitioned at 100 m intervals, and their PCI values from 2016 to 2023 were analyzed. As illustrated in Figure 6, most road sections had PCI values between 80 and 100. The road conditions were good in 2016, but there were varying degrees of damage in subsequent years. The PCI values were relatively concentrated in 2018 and 2019, while in 2020, due to large-scale maintenance activities and special weather conditions, the PCI values significantly decreased. There were also some outliers in other years. To ensure the quality of the dataset, it is necessary to consider and remove any anomalies in the data.

**Figure 7:**
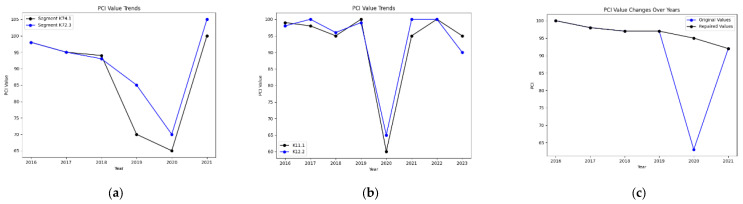
Pavement PCI data: (**a**) retained segments with natural decay patterns, (**b**) excluded segments with outliers, (**c**) interpolation for anomalous PCI values.

Segments exhibiting post-maintenance PCI recovery that aligned with natural decay patterns (e.g., K71.4 and K72.3 in Figure 7a) were retained. Conversely, segments with erratic fluctuations or outliers (e.g., K11.1 and K12.2 in Figure 7b) were removed, resulting in 1200 valid segments. For anomalous PCI values caused by maintenance or external factors (e.g., K10.6 in 2020, Figure 7c), linear interpolation using adjacent year averages was applied to reconstruct realistic decay trends (Note: K11.1 and other labels are commonly used for station identification in highway engineering to accurately locate specific locations or features of highways.). K represents kilometers, the number represents the distance from the starting point of the highway in kilometers, and the first decimal place represents the distance in hundreds of meters. For example, K71.4 represents a distance of 71 km and 400 m from the starting point, while K72.3 represents a distance of 72 km and 300 m from the starting point.

**Figure 8:**
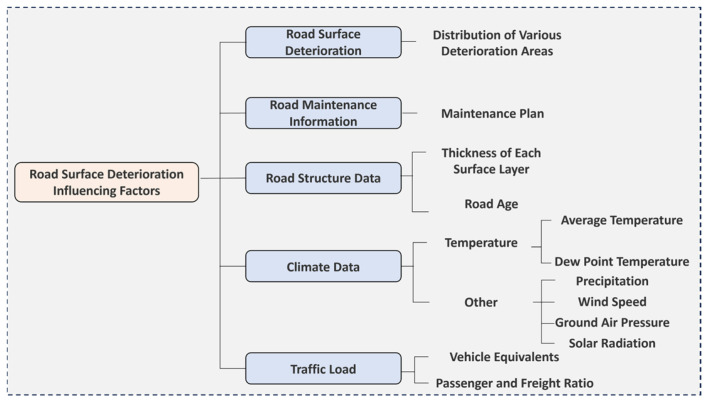
Classification of pavement deterioration influencing factors.

This study further evaluates the impacts of pavement structure, climatic conditions, traffic load, and damage types on pavement deterioration. The main influencing factors are shown in Figure 8.

As shown in Figure 8, influencing factors are categorized into five domains based on global studies and expert consultations [32]—pavement structure, meteorological factors, traffic load, damage types, and maintenance history.

### 3.2. Analysis of Factors Influencing Multi Source Features

In pavement deterioration analysis, redundant features in multi-source data can increase model complexity and reduce computational efficiency. To address this challenge, Principal Component Analysis (PCA) [33] was employed to reduce dimensionality, particularly for annual climatic and traffic-related features. PCA processes 7-year historical data, including indicator data and multi-source influencing factors such as climate and traffic features. The output of PCA is referred to as “representative annual components”, which are principal components derived from dimensionality reduction. These components are linear combinations of the original features and are designed to capture the majority of the variance in the data, effectively representing the primary patterns of variation. This process condenses the 7-year historical data into representative annual components, which serve as a streamlined yet informative basis for further analysis.

Following dimensionality reduction, a hybrid methodology combining BP-MIV and RF-RFECV was implemented to mitigate single-method bias and enhance robustness. The workflow entails three phases.

#### 3.2.1. BP-MIV Method

The BP neural network was integrated with the MIV algorithm to quantify the average impacts of input features on output predictions. Feature importance weights $w_{i}$ were calculated as

(17) $$w_{i}=\frac{\left|MIV_{i}\right|}{\sum_{k=1}^{n}\left|MIV_{k}\right|}$$

(18) $$MIV_{i}=\frac{1}{m}\sum_{j=1}^{n}IV_{i}(j),i=1,2,\ldots ,n$$

where $MIV_{i}$ represents the Mean Impact Value of the *i*-th feature. Weights were normalized to sum to 1 for cross-method comparability.

#### 3.2.2. RF-RFECV Method

RF was used to assess feature contribution, while RFECV iteratively removed non-informative features. The workflow included the following: initializing all features and training RF models; ranking features by importance scores; recursively eliminating low-importance features until reaching the optimal feature count; validating performance via cross-validation to select the final feature subset.

To enhance robustness, a weighted fusion strategy was implemented. Importance scores from BP-MIV and RF-RFECV were normalized and summed, with total weights scaled to 2. This approach balanced methodological strengths and improved generalizability.

Then, Spearman’s rank correlation coefficient [34] was applied to the top 24 candidate features. Features with high correlations were excluded. Final feature selection combined statistical significance, expert consultation, and domain knowledge.

**Figure 9:**
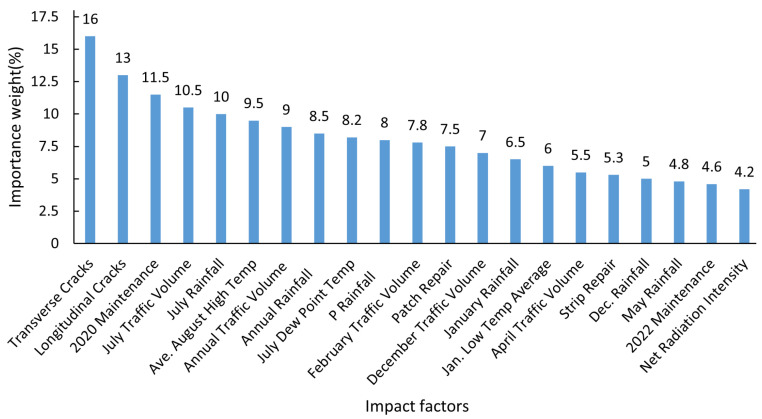
Importance ranking derived from fused BP-MIV and RF-RFECV weights.

Finally, based on the feature importance and correlation analysis, and with reference to expert opinions, the following 21 influencing factors were selected as important feature factors for pavement damage conditions, as shown in Figure 9. In addition, Table 3 provides explanations for the abbreviations of the influencing factors that appear in Figure 9.

Figure 9 illustrates the importance rankings of diverse factors affecting pavement performance based on the weighted fusion of BP-MIV and RF-RFECV methods. The importance weights exhibit a progressive decline, reflecting the factors’ varying impacts. Key factors like Transverse Cracks, Longitudinal Cracks, and 2020 Maintenance show the greatest importance weights, surpassing 10%. These factors substantially influence pavement performance. Traffic and climate-related features, including July Traffic Volume and July Rainfall, also hold significant positions. The analysis emphasizes considering these factors for comprehensive pavement performance evaluation and PCI prediction.

## 4. Comparison and Analysis of Results

### 4.1. Model Training Software and Hardware Environment

To train the BO-DLFF model, the hardware environment consists of a 12th generation Intel^®^ Core™ i5-12500 CPU running at 3.00 GHz. The development environment used a Windows 1164-bit system with an integrated development environment, Anaconda 2.3.2 and Jupyter 6.0.1. The programming language used was Python 3.6, and certain machine learning libraries utilized the Tensorflow 2.1.0 framework. In addition, Keras 2.2.4 under Tensorflow 2.1.0 was utilized as a deep neural network framework.

### 4.2. Model Performance Evaluation Metrics

The advantages and disadvantages of the prediction models are analyzed from a mathematical point of view by comparing the prediction results of the models with the actual sample values. For regression prediction tasks, R^2^, mean absolute error (MAE), root mean square error (RMSE), mean absolute percentage error (MAPE) and coefficient of determination are often used as indicators to evaluate the results.

The target pavement deterioration condition index, PCI, of this study is a regression problem, so R^2^, MAE, RMSE and MAPE are chosen as the bases for model merit judgment. The evaluation indexes of each prediction model are calculated as shown in Equations (19)–(22).

(19) $$R^{2}=1-\frac{\sum_{i=1}^{n}{\left(PCI_{i,pred}-PCI_{i,act}\right)}^{2}}{\sum_{i=1}^{n}{\left(\bar{PCI_{act}}-PCI_{i,act}\right)}^{2}}$$

(20) $$MAE=\frac{1}{n}\sum_{i=1}^{n}\left|PCI_{i,pred}-PCI_{i,act}\right|$$

(21) $$RMSE=\sqrt{\frac{1}{n}\sum_{i=1}^{n}{\left(PCI_{i,pred}-PCI_{i,act}\right)}^{2}}$$

(22) $$MAPE=\frac{100\%}{n}\sum_{i=1}^{n}\left|\frac{PCI_{i,pred}-PCI_{i,act}}{PCI_{i,act}}\right|$$

where $PCI_{i,act}$ represents the true value of PCI for the 100 m section; $\bar{PCI_{act}}$ represents the overall mean value of PCI in the sample; $PCI_{i,pred}$ is the model’s prediction of PCI for the 100 m section; n represents the total number of samples.

### 4.3. Results Analysis

The dataset was partitioned into training and test sets at an 8:2 ratio. BO was employed to tune hyperparameters. Performance was evaluated using R^2^, MAE, MSE, and MAPE metrics to derive the optimal BO-DLFF model.

#### 4.3.1. BO-DLFF Model Performance

**Figure 10:**
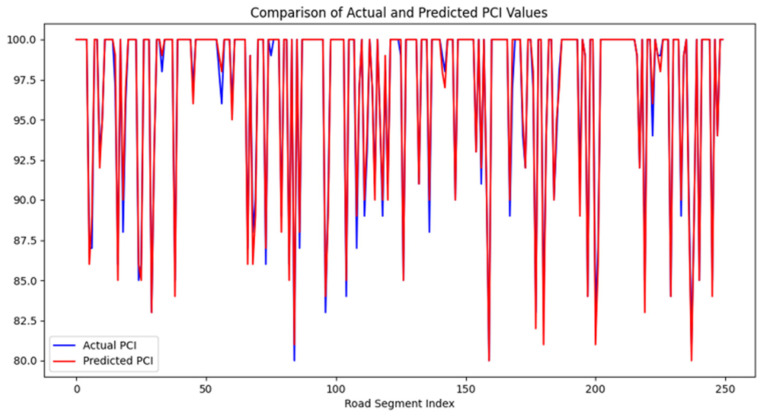
PCI prediction results of the BO-DLFF model.

The stacking strategy enhanced generalization by balancing model diversity and mitigating overfitting risks inherent to single-model approaches. Figure 10 illustrates the BO-DLFF model’s PCI prediction performance on the test set. The fusion model demonstrated robust adaptability across all road segments, effectively capturing both extreme PCI values (e.g., minima/maxima) and mitigating the localized anomalies observed in standalone models.

As can be seen from Figure 10, the constructed fusion model performs well on the test set, avoids the abnormal interference of a specific road section brought by the previous single model, ensures that it has a good performance in each road section, and achieves good prediction for both extremely large and extremely small values of PCI, presenting a more reliable and stable fitting effect. The model’s ability to integrate asynchronous sensor data streams, such as high-frequency strain measurements and traffic load profiles, significantly contributes to its robust performance across varying pavement conditions.

#### 4.3.2. Comparative Analysis of Predictive Models

To validate performance improvements, R^2^, MAE, MSE, and MAPE were compared across multiple models (Table 4). R^2^ values exceeding 0.8 indicate reliable predictive capability, as the models effectively capture PCI variation trends.

**Figure 11:**
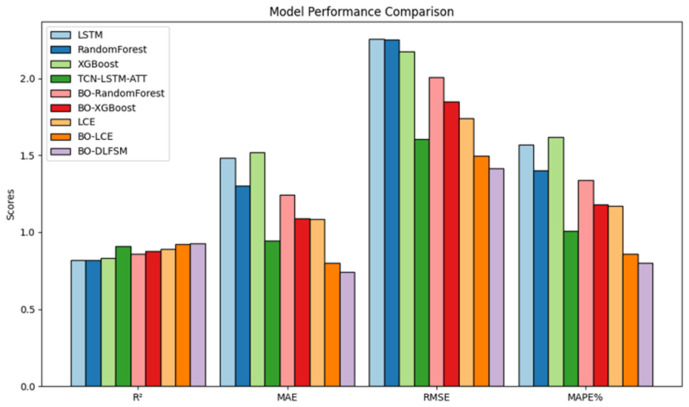
Comparison of the performances of various pavement damage prediction models.

To enhance the clarity of experimental results, key performance metrics of prominent models have been visualized for comparative analysis, as illustrated in Figure 11.

As shown in Table 4, the proposed TCN-LSTM-ATT hybrid neural network achieved an R^2^ of 0.9091 on the test set, outperforming the standalone LSTM model by 8.84%. Its error metrics (MAE, 0.9457; RMSE, 1.6055; MAPE, 1.01%) were consistently lower than those of LSTM, confirming its superior ability to capture spatiotemporal dependencies in PCI data and model non-temporal features. The integration of multi-source sensor data provided high-frequency, spatially distributed data streams, enabling the model to capture dynamic pavement responses and environmental influences more effectively. Ensemble methods, particularly the LCE model, exhibited strong performance. The baseline LCE model achieved an R^2^ of 0.8931, surpassing BO-optimized RF and XGBoost. After Bayesian Optimization, the R^2^ improved to 0.9211. The proposed BO-DLFF model further enhanced generalization, achieving the lowest errors (MAE, 0.7812; RMSE, 1.4321; MAPE, 0.82%) despite marginal R^2^ gains over BO-LCE, validating its robustness for intelligent pavement maintenance.

Compared with all the models mentioned above, the BO-DLFF model proposed in this study has been improved in its overall prediction effectiveness. Although its R^2^ value improves less notably relative to the second-ranked LCE model, the error metrics such as MAE, RMSE and MAPE are all reduced, which indicates that the BO-DLFF model excels in terms of generalization ability and stability. Its superior performance in predicting the change trend of pavement deterioration, leveraging the fusion of multi-source sensor data, provides a solid theoretical foundation for intelligent and digital pavement maintenance management.

#### 4.3.3. Ablation Studies

- Ablation Study 1: Rationale for LCE Model Selection

To validate the LCE framework’s efficacy, multiple ensemble models were evaluated on PCI prediction (Table 5). The results reveal that LCE’s hybrid Boosting-Bagging architecture consistently outperformed conventional ensemble methods (e.g., AdaBoost, Gradient Boosting) in balancing bias–variance trade-offs, particularly under sparse or noisy data conditions.

**Figure 12:**
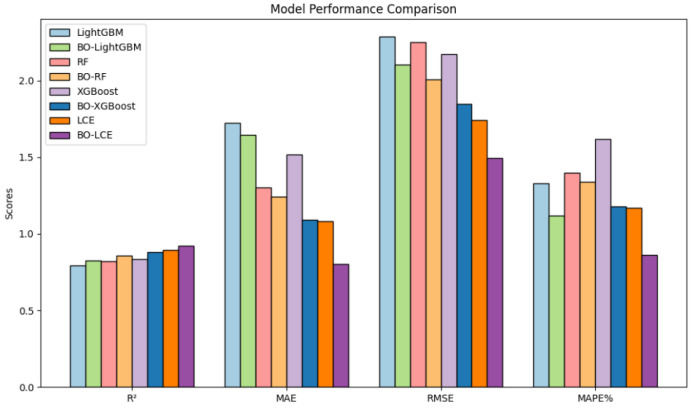
Performance comparison of various ensemble learning models.

Based on the model evaluation indexes shown in Table 4, in order to present the experimental results more clearly, the data of the model indexes with better performances were selected and visualized for comparison, as shown in Figure 12.

Table 5 presents the evaluation metrics of various models for predicting the pavement deterioration condition indicator (PCI). These models leverage data collected from an array of sensors deployed across the pavement surface and within the road structure that enable the models to capture the dynamic characteristics of pavement deterioration.

The Bayesian optimized models generally outperform their non-optimized counterparts. Among them, the BO-XGBoost model achieves the highest R^2^ of 0.8795, with an MAE of 1.0920, an RMSE of 1.8483, and an MAPE of 1.18%, indicating its superior performance in predicting PCI. The LCE model, combining XGBoost and RF through integrated learning, further enhances the prediction accuracy and stability. The unparameter-optimized LCE model already surpasses individual XGB and RF models, achieving an R^2^ of 0.8931, an MAE of 1.0842, an RMSE of 1.7405, and an MAPE of 1.17%. After Bayesian optimization, the BO-LCE model reaches an impressive R^2^ of 0.9211, an MAE of 0.8027, an RMSE of 1.4956, and an MAPE of 0.86%, outperforming all other models.

The integration of sensor data and advanced machine learning models represents a significant advancement in pavement management systems. The sensor data provide high-resolution and multi-dimensional information, capturing the complex interactions between environmental factors and pavement materials. These detailed data allow the models to identify subtle patterns and early signs of deterioration that might be missed by conventional inspection methods. The LCE model’s exceptional performance demonstrates the value of combining diverse modeling techniques with comprehensive sensor data. This integration not only improves the prediction accuracy of PCI, but also offers a more nuanced understanding of pavement deterioration mechanisms. Consequently, it provides a robust foundation for optimizing road inspection schedules, prioritizing maintenance interventions, and allocating resources efficiently, ultimately leading to enhanced road safety and extended pavement lifespan.

- Ablation Study 2: Exploring the Role of Each Component in the TCN-LSTM-ATT Module

In order to verify the prediction effect of the TCN-LSTM-ATT method on the pavement damage condition index in this study, the TCN module and the attention mechanism are discarded separately through the experiments, and the results of the model are evaluated in relation to the index of pavement damage on the pavement condition index test machine under the same data allocation conditions, as shown in Table 6.

Table 6 presents the experimental results of the prediction performances of different model configurations for the pavement deterioration condition index. The LSTM model, which relies on sequential data from pavement sensors, demonstrates relatively low prediction performance with an R^2^ of 0.8207 and an MAPE of 1.57%. The addition of the Temporal Convolutional Network (TCN) module enhances the model’s ability to capture local temporal patterns in the sensor data, improving the R^2^ to 0.8397 and reducing the MAPE to 1.42%. This indicates that TCN effectively complements LSTM in learning different pavement impact features from the sensor data. In Experiment 3, the attention mechanism is incorporated to focus on critical features within the sensor data sequences. This modification proves more effective than the TCN enhancement alone, further improving prediction performance. The combined model, integrating LSTM, TCN, and the attention mechanism, achieves the best performance with an R^2^ of 0.9091 and an MAPE of 1.01%.

The integration of these model structures enables the better capture of feature relationships in the sequential sensor data, leading to more accurate predictions of pavement deterioration. This advancement highlights the importance of combining sensor data with sophisticated machine learning architectures for improved pavement management systems. The sensor data provide high-resolution temporal information, which the enhanced LSTM model leverages to identify subtle deterioration patterns. This integration not only improves prediction accuracy, but also offers deeper insights into pavement deterioration mechanisms, supporting proactive maintenance strategies and resource optimization.

#### 4.3.4. Example Validation

The pavement damage prediction model method has better generalizability and reliability, and the pavement data of the SH highway in Hebei are selected for further validation.

The data source is a highway in Hebei with a total length of more than 200 km, covering eight districts and counties. The data characteristics include the location of the section, the roadbed width, the road’s age, construction time, investment finalization, the annual average daily traffic volume, vehicle equivalent, the axle load number, the passenger/cargo ratio, annual rainfall, air temperature, and historical breakage data in multiple dimensions, which are highly representative and comprehensive. In order to compare the prediction effects of different models on the pavement breakage condition, they are evaluated as indicators on the Hebei SH highway pavement data test set, and the results are shown in Table 7.

From Table 7, it can be seen that the fusion model in this study offers a better prediction of the pavement condition index compared to the monolithic model. Specifically, the R^2^ value of the BO-DLFF model reaches 0.9035, which is significantly higher than the comparison model. In addition, the MAE, RMSE, and MAPE of the BO-DLFF model are 0.1257, 0.1596, and 1.07%, respectively, which are lower than those of the other models, again verifying that the proposed model has a better prediction and generalization ability.

The integration of multi-source sensor data, including strain measurements and environmental parameters, enables the BO-DLFF model to achieve superior performance in capturing the complex relationships between pavement deterioration and influencing factors. This highlights the importance of leveraging sensor-based insights for accurate and reliable pavement condition assessment, ultimately supporting more informed decision-making in infrastructure maintenance and management.

## 5. Conclusions

This study presents a dual-layer fusion model (BO-DLFF) that integrates neural networks (TCN-LSTM-ATT) and ensemble learning (LCE) for predicting the pavement deterioration condition index (PCI). The model leverages data from various pavement sensors, such as ground-penetrating radar and fiber optic sensors, which provide detailed information on pavement structure and allow the real-time monitoring of environmental impacts.

The LCE component of the model effectively captures nonlinear patterns in the sensor data, while the TCN-LSTM-ATT component addresses temporal dependencies, enabling the model to learn from sequential data and identify trends in pavement deterioration. The stacking fusion technique enhances the model’s robustness, resulting in an R^2^ of 0.9292. Ablation studies confirm the necessity of each module, with significant performance gains attributed to the BO-LCE and attention mechanisms.

External validation using highway data from Hebei demonstrates the model’s superior generalizability, achieving an R^2^ of 0.9035. This indicates that the model can reliably predict PCI across different pavement conditions and sensor configurations. However, the study acknowledges limitations in exploring intrinsic feature interactions across various pavement conditions, suggesting that future work should investigate pavement-type-specific degradation patterns to further refine PCI prediction.

Overall, this framework offers a scalable and sophisticated solution for data-driven pavement maintenance, aligning with the goals of smart infrastructure management. By integrating sensor data with advanced machine learning architectures, the BO-DLFF model not only improves prediction accuracy, but also provides deeper insights into pavement deterioration mechanisms. This enables proactive maintenance strategies and optimized resource allocation, and ultimately contributes to enhanced road safety and extended pavement lifespan.

## Figures and Tables

**Table 1:** Optimal parameter values for the model.

Learner	Parameter Values
LCE	*n*_estimators = 224, max_features = 27, max_depth = 9
TCN-LSTM-ATT	filters = 32, kernel_size = 3 × 3, neurons = 50 batchsize = 64, learn_rate = 0.001, epochs = 100

**Table 2:** Data feature table.

Feature Category	Feature Name	Description	Data Type	Time Granularity	Spatial Granularity
Road Basic Info	Road Age	Number of years since the road was built and put into service	Numerical	Year	100 m Road Section
	Layer Thickness	Thickness of each layer of the pavement (upper, middle, lower layers)	Numerical	Static	100 m Road Section
Climate Data	Monthly Max Temp Mean	Average of the highest temperature each month	Numerical	Month	100 m Road Section
	Monthly Min Temp Mean	Average of the lowest temperature each month	Numerical	Month	100 m Road Section
	Annual Avg Temperature	Average temperature for each year	Numerical	Year	100 m Road Section
	Annual Dew Point Temp	Average dew point temperature for each year	Numerical	Year	100 m Road Section
	Monthly Rainfall	Rainfall for each month	Numerical	Month	100 m Road Section
	Annual Rainfall	Total rainfall for each year	Numerical	Year	100 m Road Section
Traffic Data	Monthly Vehicle Equiv	Equivalent number of vehicles for each month	Numerical	Month	100 m Road Section
	Monthly Passenger Ratio	Ratio of passenger vehicles to freight vehicles each month	Numerical	Month	100 m Road Section
	Annual Vehicle Equiv	Equivalent number of vehicles for each year	Numerical	Year	100 m Road Section
Pavement Damage	Longitudinal Crack Area	Area of longitudinal cracks per 100 m section	Numerical	Static	100 m Road Section
	Transverse Crack Area	Area of transverse cracks per 100 m section	Numerical	Static	100 m Road Section
	Block Crack Area	Area of block cracks per 100 m section	Numerical	Static	100 m Road Section
	Pothole Area	Area of potholes per 100 m section	Numerical	Static	100 m Road Section
Maintenance Data	Maintenance History	Historical maintenance records, including maintenance years and methods	Categorical	Year	100 m Road Section

**Table 3:** Explanation of impact factors.

Feature Factors	Explanation
Transverse Cracks	Cracks across the traffic direction.
Longitudinal Cracks	Cracks along the traffic direction.
2020 Maintenance	Road maintenance in 2020.
July Traffic Volume	Standardized traffic volume in July.
July Rainfall	Rainfall in July.
Ave. August High Temp	Average highest temperature in August.
Annual Traffic Volume	Annual total traffic volume.
Annual Rainfall	Annual rainfall and durability assessment.
July Dew Point Temp	Dew point temperature in July.
P Rainfall	Rainfall in a specific period.
February Traffic Volume	Traffic volume in February.
Patch Repair	Repairing diseases and restoring smoothness.
December Traffic Volume	Year-end traffic and flow impact.
January Rainfall	Winter climate and rainfall.
Jan. Low Temp Average	Low temperature and cracking risk.
April Traffic Volume	Traffic volume in April.
Strip Repair	Repair of strip-shaped damaged areas.
Dec. Rainfall	Rainfall in December.
May Rainfall	Spring climate and rainfall.
2022 Maintenance	Road maintenance in 2022.
Net Radiation Intensity	Net radiation difference.

**Table 4:** Performance comparison of pavement deterioration prediction models.

Model	R^2^	MAE	RMSE	MAPE
LSTM	0.8207	1.4827	2.2545	1.57
RF	0.8211	1.3017	2.2521	1.40
XGBoost	0.8334	1.5191	2.1736	1.62
TCN-LSTM-ATT	0.9091	0.9457	1.6055	1.01
LCE	0.8931	1.0842	1.7405	1.17
BO-RF	0.8579	1.2438	2.0069	1.34
BO-XGBoost	0.8795	1.0920	1.8483	1.18
BO-LCE	0.9211	0.8027	1.4956	0.86

**Table 5:** Performance of ensemble learning variants.

Model	R^2^	MAE	RMSE	MAPE
LightGBM	0.7916	1.7235	2.2853	1.33
BO-LightGBM	0.8268	1.6467	2.1051	1.12
RF	0.8211	1.3017	2.2521	1.40
BO-RF	0.8579	1.2438	2.0069	1.34
XGBoost	0.8334	1.5191	2.1736	1.62
BO-XGBoost	0.8795	1.0920	1.8483	1.18
LCE	0.8931	1.0842	1.7405	1.17
BO-LCE	0.9211	0.8027	1.4956	0.86

**Table 6:** Comparison of ablation experiment results.

Model	TCN	LSTM	Attention	R^2^	MAPE
Experiment 1	×	√	×	0.8207	1.57
Experiment 2	√	√	×	0.8397	1.42
Experiment 3	×	√	√	0.8782	1.18
Ours	√	√	√	0.9091	1.01

Note: √ and × indicate that the model was or was not used in the corresponding experiment, respectively.

**Table 7:** Comparison of model performance based on SH highway.

Model	R^2^	MAE	RMSE	MAPE
RF	0.8267	0.1568	0.2047	1.59
XGBoost	0.8574	0.1423	0.1869	1.36
TCN-LSTM-ATT	0.8761	0.1357	0.1782	1.24
LCE	0.8912	0.1308	0.1675	1.18
BO-DLFF	0.9035	0.1257	0.1596	1.07

## Data Availability

Data are contained within the article.
